# Radiologic and Pathologic Insights in Combined Hepatocellular–Cholangiocarcinoma: A Report of Three Cases

**DOI:** 10.3390/reports8030142

**Published:** 2025-08-08

**Authors:** Katrīna Marija Konošenoka, Nauris Zdanovskis, Aina Kratovska, Artūrs Šilovs, Veronika Zaiceva

**Affiliations:** 1 Department of Radiology, Riga Stradins University, Dzirciema Street, 16, LV-1007 Riga, Latvia; 036336@rsu.edu.lv (K.M.K.); aina.kratovska@aslimnica.lv (A.K.); arturs.silovs@rsu.lv (A.Š.); 2 Department of Interventional Radiology, Riga East University Hospital, Hippocrates St, 2, LV-1038 Riga, Latvia; veronika.zaiceva@aslimnica.lv

**Keywords:** combined hepatocellular–cholangiocarcinoma, liver tumor biopsy, hepatocellular carcinoma, intrahepatic cholangiocarcinoma, liver MRI, immunohistochemistry

## Abstract

**Background and Clinical Significance**: Combined hepatocellular–cholangiocarcinoma (cHCC-CC) is a rare primary liver malignancy exhibiting both hepatocellular and cholangiocellular features. Due to overlapping clinical, imaging, and pathological characteristics with hepatocellular carcinoma (HCC) and intrahepatic cholangiocarcinoma (iCC), diagnosis remains challenging. Early and accurate differentiation is critical for optimal treatment planning. **Case Presentation**: We report three histologically confirmed cases of cHCC-CC with different imaging features, biomarker profiles, treatment strategies, and clinical outcomes. Patient 1, a 69-year-old female, presented with a large centrally located liver mass exhibiting iCC-like imaging features and mildly elevated AFP and CA 19-9 levels. Biopsy confirmed poorly differentiated cHCC-CC. Treatment involved palliative chemotherapy, with a survival of 16 months following diagnosis. Patient 2, an 80-year-old female with a small lesion in a cirrhotic liver, demonstrated an HCC-like enhancement pattern but normal AFP levels. Surgical resection was performed, and histology confirmed cHCC-CC with a dual phenotype. Despite initial remission, intrahepatic recurrence developed, treated with TACE and systemic therapy. The patient later transitioned to palliative care due to progression and survived 36 months. Patient 3, a 67-year-old male with chronic hepatitis C, presented with an HCC-like lesion and elevated AFP. Due to comorbidities, surgical resection was not feasible, and the patient was treated with percutaneous microwave ablation as a safer alternative. Biopsy during ablation confirmed cHCC-CC; follow-up was ongoing at submission. **Conclusions**: These cases highlight the diagnostic complexity and clinical variability of cHCC-CC. Imaging may be misleading, and tumor markers do not reliably predict subtype or prognosis. Histological confirmation is essential, particularly in patients with atypical imaging or discordant biomarker profiles. Individualized management, informed by tumor biology and patient condition, remains critical. Further research is needed to refine diagnostic criteria and develop tailored therapeutic strategies for this challenging tumor entity.

## 1. Introduction and Clinical Significance

Primary liver cancer is the sixth most diagnosed malignancy and the third leading cause of cancer-related mortality worldwide, with hepatocellular carcinoma (HCC) and intrahepatic cholangiocarcinoma (iCCA) representing the most prevalent subtypes [1,2,3].

Combined hepatocellular carcinoma and cholangiocarcinoma (cHCC-CC) is a rare and heterogeneous malignancy that exhibits histological features of both hepatocytic and cholangiocytic differentiation within the same tumor [4,5].

Representing less than 5% of all primary liver cancers, cHCC-CC presents considerable diagnostic and therapeutic challenges because of overlapping radiological features with HCC and iCCA, and the absence of specific serum biomarkers [6,7,8,9].

On imaging, cHCC-CC may mimic either HCC or iCCA, depending on the dominant phenotype, often leading to initial misclassification. Radiologic uncertainty is further complicated by the variability in vascular behavior, including arterial phase hyperenhancement (APHE), venous washout, rim enhancement, and restricted diffusion, features that are variably shared with both HCC and iCC [10,11].

In the absence of definitive radiologic features, tissue diagnosis remains essential for accurate classification. Histopathologic confirmation, supported by immunohistochemical staining, is necessary to establish biphenotypic differentiation, guiding appropriate therapeutic strategies and prognostication [9,12,13].

Recent molecular studies suggest that cHCC-CC may arise from a common hepatic progenitor cell capable of bipotential differentiation. Clonal genetic alterations shared between the hepatocytic and cholangiocytic components support this theory, reinforcing the concept of molecular and histological heterogeneity within a single neoplasm [14,15,16]. The 2019 World Health Organization (WHO) classification now defines cHCC-CC based primarily on morphology [5].

From an etiological perspective, cHCC-CC shares risk factors with both HCC and iCC, including chronic hepatitis B or C, alcohol-related liver disease, cirrhosis, and non-alcoholic fatty liver disease [5,8,17,18,19]. Clinically, it often presents with non-specific symptoms, such as right upper quadrant discomfort, weight loss, or fatigue.

Biomarkers such as alpha-fetoprotein (AFP) and carbohydrate antigen 19-9 (CA 19-9) may be variably elevated but are not reliable for diagnosis [8,9,19]. Co-elevation of AFP and CA 19-9 may suggest cHCC-CC, but such patterns lack specificity [20].

Despite advances in imaging—including hepatobiliary contrast-enhanced MRI and diffusion-weighted imaging (DWI)—preoperative diagnosis of cHCC-CC remains elusive in many cases. The Liver Imaging Reporting and Data System (LI-RADS) offers a standardized approach to characterizing hepatic lesions in at-risk patients; however, up to 50% of cHCC-CC cases may be misclassified as either typical HCC (LR-5) or probably malignant but not HCC-specific (LM-M) [21,22,23]. These limitations necessitate histological confirmation in ambiguous or non-HCC-typical presentations.

The prognosis in cHCC-CC patients is associated with poorer outcomes compared with conventional HCC, and its clinical course tends to be more aggressive. While some studies report overall survival rates similar to intrahepatic cholangiocarcinoma, others suggest intermediate survival between HC and iCC subtypes [24,25,26].

The five-year survival rate after surgical resection ranges from 20% to 40%, and recurrence is common, particularly in cases with a dominant cholangiocytic component [24,27,28]. The presence of vascular invasion, lymph node metastasis, and a cholangiolar phenotype has been associated with worse cancer-specific survival [24,29]. Given its aggressive behavior and frequent resistance to standard therapies, cHCC-CC remains a therapeutic challenge.

In this report, we present three histologically confirmed cases of cHCC-CC with distinct clinical presentations and imaging profiles. These cases underscore the limitations of radiological assessment in predicting tumor biology, especially when lesions mimic typical HCC or iCC. Through detailed radiopathologic correlation, we aim to demonstrate key diagnostic pitfalls and therapeutic challenges that clinicians may encounter in practice.

Given the diagnostic uncertainty, imaging overlap, and absence of standardized treatment protocols, case-based clinical evidence remains essential to enhance clinical awareness and guide decision-making. The presented cases illustrate the phenotypic diversity, radiologic ambiguity, and biological complexity of cHCC-CC, highlighting the importance of maintaining a high index of suspicion when imaging findings are discordant or non-specific. By contributing to a growing body of literature, our report supports early multidisciplinary involvement and histological confirmation as critical steps toward accurate diagnosis, timely treatment, and improved patient outcomes in this rare malignancy.

## 2. Case Presentation

To contextualize the following case presentations, we briefly summarize the common diagnostic and therapeutic approaches applied across all patients. Each case underwent a multimodal diagnostic evaluation, including contrast-enhanced multiphase CT and MRI with hepatobiliary contrast agents and diffusion-weighted imaging (DWI). Histopathological confirmation was obtained via liver biopsy or surgical specimen, with immunohistochemistry performed to assess tumor differentiation using markers such as CK7, CK19, CK20, CK AE1/AE3, glypican-3, TTF-1, and CD34. Serum tumor markers, including AFP, CA 19-9, and CEA, were evaluated to aid differential diagnosis. Treatment strategies varied according to clinical presentation and imaging features, and included surgical resection, transarterial chemoembolization (TACE), and systemic therapy. Radiologic findings were interpreted using the LI-RADS criteria, with diagnoses confirmed through multidisciplinary consensus [30].

### 2.1. Case 1: iCC-like Lesion Treated with Palliative Chemotherapy

A 69-year-old female patient presented with a three-month history of intermittent diarrhea and unintentional weight loss of approximately 12 kg. She had a background of type 2 diabetes mellitus, class I pulmonary arterial hypertension, and prior left nephrectomy due to nephrolithiasis.

Tumor marker analysis showed a mildly elevated alpha-fetoprotein (AFP) level of 14 ng/mL and carbohydrate antigen 19-9 (CA 19-9) of 56 U/mL, while carcinoembryonic antigen (CEA) was not reported.

**Figure 1:**
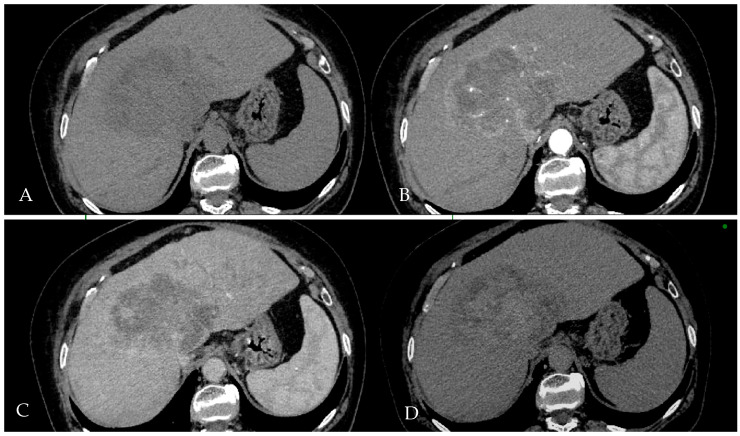
Multiphasic contrast-enhanced CT of Patient 1 shows an iso- to mildly hypodense lesion on non-contrast (**A**), with peripheral arterial enhancement (**B**), followed by heterogenous centripetal enhancement in the portal and delayed phases (**C,D**). Low-density areas suggest necrosis or fibrosis. The imaging lacks typical LI-RADS 5 features (e.g., non-rim APHE or capsule), and the pattern is consistent with LR-M, indicating probable malignancy not specific for HCC.

Initial contrast-enhanced abdominal CT imaging was performed as a part of the initial imaging, which revealed a large, lobulated hepatic mass measuring approximately 12 × 9 × 11 cm (Figure 1). The lesion was centrally located, involving segments 4a, 4b, 5, 8, and the caudate lobe (S1), with accompanying moderate intrahepatic cholestasis, along with periportal, paraaortic, and paracaval lymphadenopathy, and small-volume ascites. The radiological differential diagnosis included intrahepatic cholangiocarcinoma and hepatocellular carcinoma.

**Figure 2:**
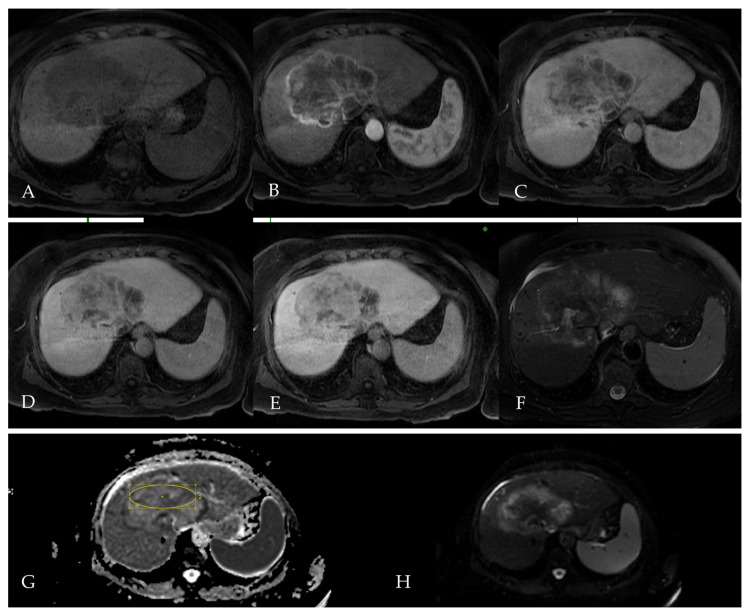
Multiphasic liver MRI, performed for further lesion characterization following CT in Patient 1. On T1-weighted pre-contrast images (**A**), the lesion appears hypointense, while on T2-weighted images (**F**), it shows heterogeneous hyperintensity, particularly in the periphery. The lesion shows rim-like arterial phase hyperenhancement (APHE, **B**), followed by progressive centripetal enhancement in the portal (**C**) and delayed (**D**) phases. In the hepatobiliary phase (**E**), the lesion remains hypointense. Diffusion-weighted imaging (DWI, **H**) shows peripheral hyperintensity, and the ADC map shows mild peripheral hypointensity (**G**).

To further characterize the lesion and guide multidisciplinary team decision-making, a liver MRI was performed several weeks later (Figure 2). The lesion, measuring up to 12 cm, exhibited rim-like arterial phase hyperenhancement (Figure 2B), followed by progressive centripetal hyperenhancement in the later portal and delayed imaging phases (Figure 2C,D). In the hepatobiliary phase (HBP), acquired 20 min after hepatospecific contrast (gadoxetate) injection, the lesion appears hypointense relative to the surrounding liver parenchyma. The lack of uptake within the lesion suggests an absence of functioning hepatocytes, a characteristic feature of non-HCC malignancies, such as cholangiocarcinoma or combined HCC-CC [31,32].

Due to the absence of an automatically generated ADC map in the original imaging protocol, apparent diffusion coefficient (ADC) values were manually calculated using the GE Healthcare imaging workstation. High b-value (b = 1000 mm^2^/s) diffusion-weighted imaging (DWI) demonstrates the peripheral hyperintensity of the lesion with a hypointense central area, consistent with viable tumor tissue at the rim and likely central necrosis or fibrosis (Figure 2H). The corresponding ADC map shows hypointense signal in the periphery, suggestive of incomplete or partial diffusion restriction (Figure 2G). The center appears relatively hyperintense, further supporting a non-viable tumor core. The mean ADC values, measured in the selected region of interest (ROI), were 1.21 × 10^–3^ mm^2^/s and a deviation of 191 × 10^–3^ mm^2^/s.

Given the presence of ancillary features as non-HCC-specific findings, the lesion was categorized as LI-RADS LR-M (probably malignant but not specific for HCC), indicating the need for histological confirmation.

A percutaneous ultrasound-guided biopsy of the hepatic lesion was performed without complications. Histopathological analysis revealed a poorly differentiated (grade 3) combined hepatocellular–cholangiocarcinoma (cHCC-CC) composed of pleomorphic epithelial cells with trabecular and glandular architecture and focal necrosis. Reticulin staining showed a preserved framework around tumor nests. Immunohistochemistry demonstrated positivity for CK AE1/AE3, and negativity for CK7, CK20, AFP, and glypican-3, confirming biphenotypic differentiation.

Due to the tumor’s central location, large size, and vascular involvement (including portal vein encasement and compression of the inferior vena cava), the lesion was deemed unresectable. A multidisciplinary tumor board recommended palliative chemotherapy, and the patient began treatment with FOLFIRINOX (oxaliplatin, leucovorin, irinotecan, and 5-fluorouracil), using reduced doses because of comorbidities. The treatment was tolerated with manageable side effects, and imaging follow-up was planned after 2–3 cycles. A follow-up contrast-enhanced CT scan showed no significant change compared with previous imaging. Despite an initially stable condition (ECOG 0-1), the disease progressed, and the patient passed away approximately 16 months after diagnosis.

### 2.2. Case 2: HCC-like Lesion Undergoing Surgical Resection and Sequential Therapies

**Figure 3:**
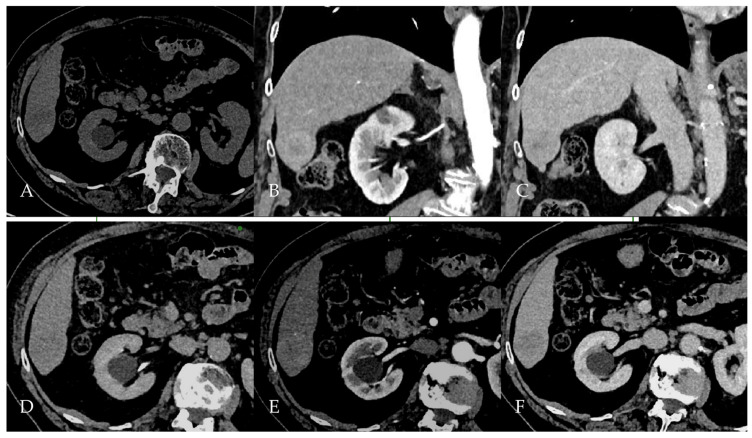
Contrast-enhanced abdominal CT scan in Patient 2. The scan reveals a 3.5 cm low-density lesion in the inferior aspect of the right hepatic lobe (**A**). The lesion demonstrates heterogeneous hyperenhancement in the arterial phase (**B**,**E**), followed by washout in the venous phase (**C**,**F**), and appears hypodense relative to the surrounding liver parenchyma on the delayed phase (**D**).

An 80-year-old female was referred for hepatobiliary evaluation following the incidental detection of a hepatic lesion on contrast-enhanced CT imaging (Figure 3). The liver did not demonstrate evident radiologic features of advanced cirrhosis, such as surface nodularity, parenchymal heterogeneity, and segmental volume redistribution. However, the patient had a documented history of cirrhosis prior to this hospitalization, suggesting an underlying chronic liver disease despite the lack of imaging evidence at the time of examination.

On multiphasic contrast-enhanced CT, the lesion measured approximately 3.5 cm and was located in the inferior aspect of the right hepatic lobe. It demonstrated non-rim arterial phase hyperenhancement (Figure 3B,E) and non-peripheral washout in the portal (Figure 3C,F) and delayed (Figure 3F) phases, two major features of hepatocellular carcinoma according to LI-RADS v2018 [30]. No enhancing capsule was clearly identified, and threshold growth could not be assessed due to a lack of prior imaging. Given the lesion’s size and enhancement pattern, and assuming an at-risk liver, these features would fulfill criteria for a LI-RADS 5 observation, indicating a high probability of HCC.

A follow-up multiphasic liver MRI was performed to further characterize the hepatic lesion initially identified on CT to support diagnostic differentiation between hepatocellular carcinoma and other primary liver malignancies prior to initiating treatment planning. The MRI was indicated prior to treatment planning in order to provide higher-resolution tissue characterization, vascular phase assessment, and functional imaging with diffusion-weighted sequences. The liver demonstrated imaging features suggestive of cirrhosis, including surface nodularity, parenchymal heterogeneity, and segmental contour changes. Mild hypertrophy of the lateral segment of the left hepatic lobe was observed.

**Figure 4:**
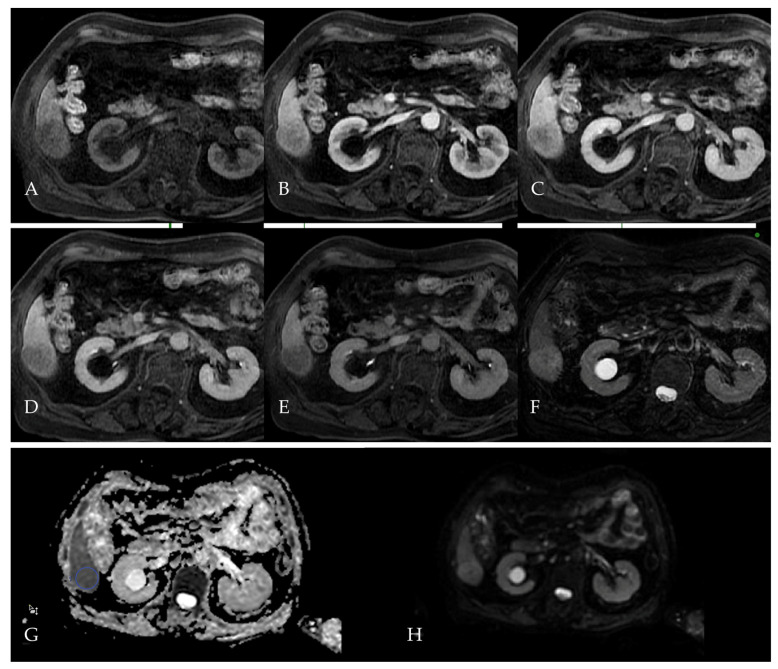
Multiphasic liver MRI, performed for further lesion characterization following CT in Patient 2. On T1-weighted pre-contrast imaging (**A**), the lesion appears hypointense, while being hyperintense on T2-weighted (**F**) imaging. The lesion demonstrates heterogeneous non-rim arterial phase hyperenhancement (APHE, **B**), followed by washout in the portal (**C**) and delayed (**D**) phases. In the hepatobiliary phase (**E**), the lesion appears hypointense. Diffusion-weighted imaging (DWI, **H**) shows peripheral hyperintensity, and the ADC map (**G**) demonstrates corresponding hypointensity.

The MRI (Figure 4) demonstrated a well-circumscribed lesion in segment VI, measuring approximately 4.5 cm, with evidence of interval growth compared with a prior CT performed two months earlier (Figure 3). The lesion showed heterogeneous arterial phase hyperenhancement (Figure 4B), followed by progressive late washout in the portal venous and delayed phases (Figure 4C,D), and a thin enhancing capsule (Figure 4C), consistent with imaging features typical of hepatocellular carcinoma. In the hepatobiliary phase (Figure 4E), acquired 20 min after administration of gadoxetate disodium, the lesion appears hypointense relative to the surrounding liver parenchyma, a finding more consistent with non-HCC malignancy or suggestive of poorly differentiated HCC. On diffusion-weighted imaging (DWI), the lesion shows restricted diffusion, appearing hyperintense on high b-value images (Figure 4H, b = 1000 mm^2^/s) and hypointense on the corresponding ADC map (Figure 4G). The mean apparent diffusion coefficient (ADC) value, measured within the selected region of interest (ROI), was 0.99 × 10^–3^ mm^2^/s with a standard deviation of 85 × 10^–3^ mm^2^/s.

Laboratory testing revealed normal alpha-fetoprotein (AFP: 3.16 ng/mL) and carcinoembryonic antigen (CEA: 1.46 ng/mL), with a mildly elevated carbohydrate antigen 19-9 (CA 19-9: 41.0 U/mL). Liver enzymes and synthetic function were within normal limits. The patient was asymptomatic, and physical performance was good (ECOG 0).

The case was evaluated by a multidisciplinary tumor board, which noted the patient’s preserved hepatic function (Child–Pugh A), Karnofsky performance score >70, and a projected survival of more than six months. Given the lesion’s imaging profile, localized nature, and the patient’s stable condition, a decision was made to proceed with curative-intent surgical resection.

The patient underwent atypical resection of segment VI. Intraoperatively, the liver had a cirrhotic appearance. A cystic tumor with necrotic areas was successfully excised. The postoperative course was uneventful. Histopathological evaluation of the resected lesion confirmed a combined hepatocellular–cholangiocarcinoma (cHCC-CC) composed of both moderately differentiated (grade 2) cholangiocarcinoma (CK7+, CK19+, and CD34+ sinusoidal vessels) and well-differentiated (grade 1) hepatocellular carcinoma (glypican-3 focal+ and TTF-1+) components. The tumor was staged as pT1bN0M0L+V-R0, with lymphatic but no vascular or perineural invasion. Resection margins were negative. The background liver tissue demonstrated macronodular changes consistent with cirrhosis, thus confirming cirrhosis histologically at this point.

**Figure 5:**
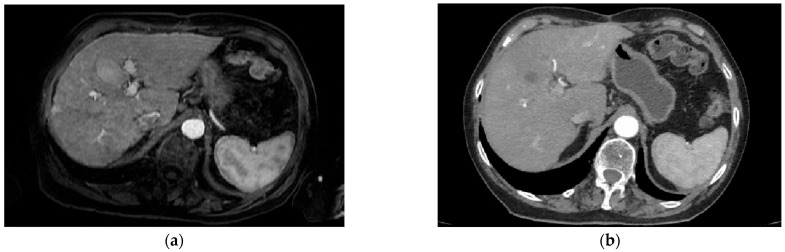
Postoperative follow-up scans of Patient 2: (**a**) liver MRI shows a new lesion in segments 4a/4b with arterial phase hyperenhancement (APHE); (**b**) follow-up CT scan performed three months after TACE demonstrates the same lesion with partial necrosis and marked reduction in arterial enhancement, indicating treatment response.

A postoperative follow-up MRI two months later (Figure 5a) revealed a new lesion in segments 4a/4b, measuring 2.7 cm, with a necrotic center and associated enlargement of the portocaval lymph node, consistent with intrahepatic recurrence and regional nodal spread. Following the multidisciplinary tumor board’s recommendation, transarterial chemoembolization (TACE) was carried out approximately four weeks afterward. The patient was also started on first-line systemic therapy with Sorafenib (800 mg/day).

A CT scan performed about one month after TACE (Figure 5b) showed partial necrosis of the treated lesion, but new small hypodense lesions appeared in both hepatic lobes. Despite ongoing Sorafenib therapy, subsequent MRIs over the following months revealed progressive intrahepatic disease and lymphadenopathy, confirming treatment resistance.

With preserved liver function and functional status, the patient was transitioned to second-line treatment with Regorafenib (160 mg/day, 21/28-day regimen). However, this therapy was poorly tolerated and failed to halt disease progression. Approximately two years after the initial diagnosis, imaging confirmed extensive disease progression, including multiple bilobar hepatic lesions, pulmonary metastases, and mediastinal and paracardiac lymphadenopathy.

By the third year after diagnosis, the patient developed symptoms of jaundice, pruritus, and right upper quadrant pain. Laboratory testing revealed hepatic decompensation, with total bilirubin 69.3 µmol/L, direct bilirubin 54.9 µmol/L, AST 296 U/L, and GGT 176 U/L. With no remaining curative options, the patient was transitioned to supportive and palliative care, including ursodeoxycholic acid, silymarin, antihistamines, and analgesics. The total disease duration was 36 months before the patient passed away.

### 2.3. Case 3: HCC-like Lesion in a Chronic Hepatitis C Patient Managed with Ablation

A 67-year-old male with a history of hepatitis C virus (HCV) infection underwent routine surveillance imaging, which revealed a hepatic lesion. His medical history was notable for multiple comorbidities, including multiple sclerosis, coronary artery disease, prior myocardial infarction, and post-percutaneous coronary intervention (PCI) status. Laboratory tests at the time showed mildly elevated liver enzymes, with ALAT at 47 U/L and ASAT at 56 U/L, while other liver function indicators—such as bilirubin, alkaline phosphatase, and albumin—were within normal limits. As part of the routine hepatology follow-up, transient elastography (FibroScan) showed a median liver stiffness of 13.9 kPa, indicating significant fibrosis. The findings reflected relatively preserved liver function in the setting of chronic viral hepatitis. Given these findings and the patient’s risk profile, cross-sectional imaging was performed to further evaluate the lesion.

**Figure 6:**
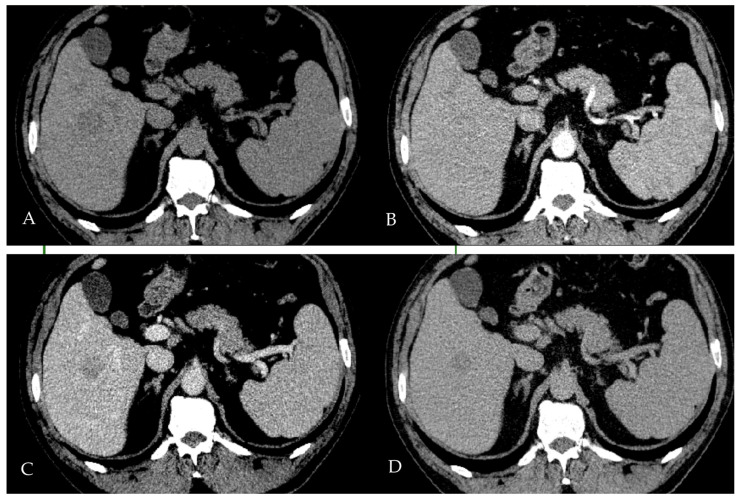
Multiphasic contrast-enhanced abdominal CT scan in Patient 3. The scan reveals a 4.2 cm hypodense lesion in segment V of the right hepatic lobe (**A**). The lesion demonstrates mostly peripheral hyperenhancement in the arterial phase (**B**), followed by washout in the portal venous phase (**C**), appearing hypodense to liver parenchyma on the delayed phase (**D**).

The multiphasic contrast-enhanced CT of the abdomen (Figure 6) revealed a well-circumscribed lesion in segment V of the liver, measuring approximately 3 × 3.3 × 4.2 cm. The lesion demonstrated mild arterial phase peripheral enhancement (Figure 6B) with subsequent washout in the portal venous phase (Figure 6C,D). These enhancement characteristics were suspicious for HCC, although a secondary lesion could not be excluded. Overall, imaging features were highly suggestive of HCC, prompting further evaluation with liver-specific MRI to refine the diagnosis and confirm lesion characterization.

**Figure 7:**
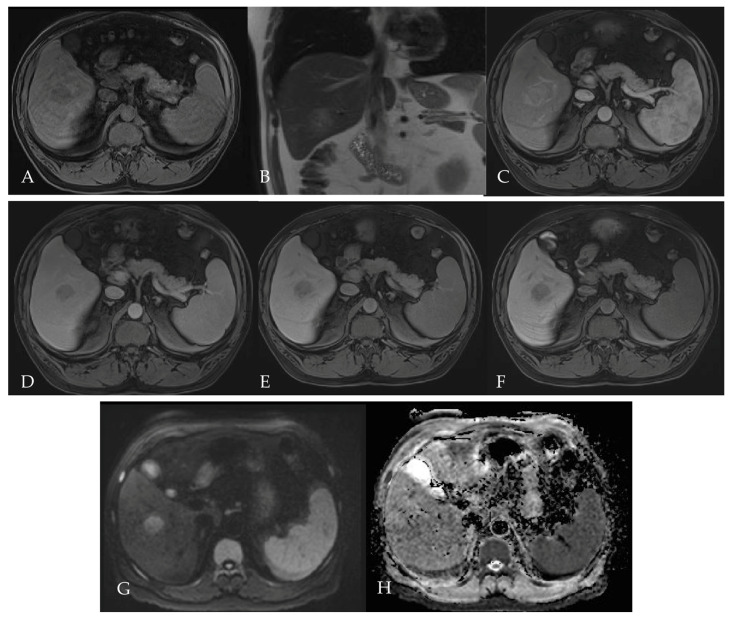
Multiphasic liver MRI, performed for further lesion characterization following CT in Patient 3. On T1-weighted pre-contrast imaging (**A**), the lesion appears hypointense, while being hyperintense on T2-weighted (**B**) imaging. The lesion demonstrates non-rim arterial phase hyperenhancement (APHE, **C**), followed by washout in the portal (**D**) and delayed (**E**) phases. In the hepatobiliary phase (**F**), the lesion appears hypointense. Diffusion-weighted imaging (DWI, **G**) shows hyperintensity, and the ADC map (**H**) demonstrates corresponding hypointensity.

A month later, the patient underwent a liver MRI for further evaluation of the suspected hepatic lesion (Figure 7). The liver was of a normal size with smooth contours. In segment V of the right hepatic lobe, a well-circumscribed lesion measuring up to 3.2 cm was identified. The lesion appeared hyperintense on T2-weighted imaging (Figure 7B), showed restricted diffusion with corresponding hypointensity on the ADC map (Figure 7G,H), and demonstrated non-rim arterial phase hyperenhancement (Figure 7C) followed by washout in the portal venous and delayed phases Figure 7D,E). The mean ADC values, measured in the selected region of interest (ROI), were 0.88 × 10^–3^ mm^2^/s and a deviation of 193 × 10^–3^ mm^2^/s. In the hepatobiliary phase (Figure 7F), the lesion was hypointense relative to the liver parenchyma. The lesion’s imaging profile is highly suggestive of hepatocellular carcinoma (HCC) and qualifies as LI-RADS 5. No additional lesions were seen elsewhere in the liver.

Although surgical resection or TACE were initially considered, the extent of resection and the patient’s significant comorbidities rendered the procedure high risk. Consequently, the multidisciplinary tumor board recommended a less invasive approach, and percutaneous microwave ablation (MWA) was selected as the preferred treatment modality. The patient subsequently underwent CT-guided MWA of a hepatic lesion in the right lobe, previously deemed likely hepatocellular carcinoma. Under general anesthesia, two core needle biopsies were obtained using an 18G TruCut needle. MWA was then performed using two 14G TATO probes, with adequate ablation confirmed on control CT, showing an appropriate safety margin around the lesion.

Histopathologic evaluation of the lesion demonstrated an infiltrative tumor with solid architecture, composed of markedly pleomorphic cells. Immunohistochemically, the tumor showed strong positivity for CK19, CK7, and CD34 in ductular–trabecular structures, as well as glypican-3 and TTF-1 in solid components. AFP staining was non-informative. These findings support a mixed phenotype with both hepatocellular and cholangiocellular differentiation.

Serum tumor markers further reflected a dominant hepatocellular component, with a significantly elevated AFP level of 100 ng/mL, while CA 19-9 and CEA remained within normal limits. These results, particularly the elevated AFP alongside cholangiocellular markers on immunohistochemistry staining, are in line with the histologically confirmed diagnosis of cHCC-CC.

The patient was discharged in stable condition with a recommendation for follow-up liver MRI at three-month intervals to evaluate treatment efficacy; imaging results were still pending at the time of manuscript submission.

To summarize data from all three clinical cases, Table 1 provides a comparative overview of imaging features, histopathologic components, immunohistochemical profiles, and tumor marker levels. This side-by-side presentation highlights both shared characteristics and diagnostic variations among the cases, facilitating a more integrated understanding of cHCC-CC presentation.

## 3. Discussion

### 3.1. Summary

Combined hepatocellular–cholangiocarcinoma (cHCC-CC) is a rare heterogeneous malignancy characterized by the presence of both hepatocytic and cholangiocytic differentiation within the same tumor, as defined in the latest World Health Organization (WHO) classification [4,5,6]. Although all patients were ultimately diagnosed with cHCC-CC, the initial clinical impressions, radiologic findings, and treatment decisions diverged significantly, reflecting the tumor’s variable phenotypic expression and clinical behavior.

Patient 1 exhibited a large hepatic mass with imaging features more suggestive of intrahepatic cholangiocarcinoma (iCC), including rim arterial hyperenhancement, centripetal contrast filling, and hypointensity on hepatobiliary phase MRI [30,33,34]. Data from the literature support that cHCC-CC in patients without classic HCC risk factors are more frequently classified as LR-M on MRI, and these tumors tend to be larger and more likely to present at an advanced stage, often precluding surgical options [35,36].

In contrast, Patient 2 presented with a small, resectable lesion in a cirrhotic liver, demonstrating classic hepatocellular carcinoma (HCC) features, including arterial hyperenhancement (non-rim), washout, and a capsule [20,30,37]. Surgery was pursued without preoperative biopsy, consistent with established HCC management protocols [20,38,39]. Upon recurrence, the patient underwent transarterial chemoembolization (TACE) and systemic therapy, as TACE is considered more effective in cHCC-CC lesions with hypervascular (HCC-like) enhancement patterns with an overall response and survival rate inferior to HCC [40,41]. Similarly, Patient 3 exhibited HCC-like imaging features, with elevated AFP (100 ng/mL) and normal CA 19-9 and CEA levels, supporting the diagnosis.

The reported cases exemplify the diagnostic complexity of cHCC-CC based on imaging findings. While some lesions exhibit dual or mixed imaging features, many mimic either HCC or iCC, often resulting in incorrect classification. Studies indicate that up to 50% of cHCC-CC are misclassified as HCC when assessed using LI-RADS criteria [21,22,23]. Hence, histological confirmation remains essential, particularly when imaging and clinical features are discordant. Immunohistochemistry and multiregional biopsies are recommended to detect biphenotypic differentiation [42].

In the broader scientific context, the 2019 WHO classification, informed by international consensus, now defines cHCC-CC based on morphology, with immunohistochemistry aiding in subtyping, and recognizes “intermediate cell carcinoma” as a separate variant. Cholangiocellular carcinoma has been reclassified under iCC. Molecular studies support a monoclonal origin for most cHCC-CC, with clonal alterations shared across hepatocytic and cholangiocytic components. However, the histology of recurrent or metastatic lesions may vary, reflecting the tumor’s biological plasticity [4,5,43].

Prognostically, cHCC-CC demonstrates outcomes intermediate between HCC and iCC. Radical resection remains the only curative modality, but five-year survival remains modest at 20–40%, and recurrence rates are high [44,45]. The dominant histological component is a key predictor of recurrence and survival. While liver transplantation has transformed outcomes in HCC, its role in cHCC-CC remains controversial because of high recurrence and lack of data.

Patient outcomes in cHCC-CC vary not only because of differences in clinical presentation and tumor biology but also as a result of divergent treatment strategies, making it challenging to determine the optimal therapeutic approach for this heterogeneous malignancy. Surgical resection is the only established curative treatment strategy for cHCC-CC and is recommended as first-line therapy when feasible, with outcomes similar to resection for HCC or iCC [20,45]. There is insufficient evidence for biphenotypic tumors to determine whether MWA is as effective as TACE, as most data and guideline recommendations focus on HCC rather than cHCC-CC [46,47]. Overall, the evidence base for locoregional therapies in cHCC-CC is much weaker than for HCC, and these modalities are generally reserved for patients who are not surgical candidates or as a part of multimodal or palliative strategies [48,49].

Therapeutically, there is no standardized systemic regimen for cHCC-CC. Treatments for HCC (e.g., Sorafenib, lenvatinib, or immunotherapy) and for iCC (e.g., gemcitabine–cisplatin) are often used, but response rates are inconsistent [50,51,52]. Future therapeutic strategies may benefit from molecular subtyping and targeted therapies, including immune checkpoint inhibitors and tyrosine kinase inhibitors, though prospective trials specifically addressing cHCC-CC remain lacking [53,54,55].

### 3.2. Implications and Future Directions

The diagnostic challenges posed by cHCC-CC emphasize the need for heightened clinical suspicion in patients with liver tumors that exhibit overlapping imaging features and non-specific biomarker profiles. The integration of advanced imaging techniques, immunohistochemistry, and molecular diagnostics is essential for accurate classification. Given the poor prognosis and lack of effective systemic therapies, future research should focus on prospective clinical trials, genomic and transcriptomic profiling, and personalized treatment strategies based on dominant cellular lineage or molecular targets. Collaborative registries and multicenter studies are also needed to improve our understanding of this rare malignancy and to guide evidence-based clinical management.

## 4. Conclusions

Combined hepatocellular carcinoma and cholangiocarcinoma (cHCC-CC) is a rare and diagnostically elusive primary liver tumor. These case reports illustrate how cHCC-CC may radiologically mimic HCC or CC on CT and MRI, including hyperenhancement and washout, while being biologically distinct.

The nature of tumor biology and biomarker expression is controversial. Tumor markers alone were not reliable indicators of tumor type or aggressiveness, and imaging characteristics, while informative, did not fully predict prognosis. Notably, patients with more HCC-like lesions had a more favorable clinical course, while the more iCC-like presentation was associated with rapid progression despite biopsy-proven diagnosis.

In all cases, diffusion restriction and discordant tumor markers raised suspicion, but only histopathological confirmation—including dual immunophenotypic staining—established the correct diagnosis. These reports underscore the clinical necessity of pursuing biopsy in liver lesions, especially with atypical imaging features, and highlight the limitations of radiology in cases where tumor biology is more complex than appearances suggest. The cases add to the growing recognition that cHCC-CC must be considered in the differential diagnosis of focal liver lesions to avoid misdiagnosis and inappropriate treatment planning.

## Figures and Tables

**Table 1:** Integrated radiologic, histopathologic, and serologic features in three cases of combined hepatocellular–cholangiocarcinoma.

Feature	Case 1	Case 2	Case 3
Arterial phase	Rim APHE	Non-rim APHE	Non-rim APHE
Venous phase	Heterogeneous centripetal enhancement	Washout, non-rim; thin enhancing capsule	Washout, non-rim
Delayed phase	Heterogeneous centripetal enhancement	Washout, non-rim	Washout, non-rim
Hepatobiliary phase	Hypointense, heterogeneous	Hypointense	Hypointense
Diffusion restriction	Moderate, partial, mostly peripheral	Moderate, diffuse, mostly peripheral	Marked, diffuse
Histological components	Mixed HCC/CC	Mixed HCC/CC	Mixed HCC/CC
AFP level	Mildly elevated	Normal	Significantly elevated
CA 19-9 level	Mildly elevated	Mildly elevated	Normal
CEA	Not reported	Normal	Normal
Immunohistochemistry markers	CK AE1/AE3+, CK7−, CK20−, Glypican-3-, AFP-	CK7+, CK19+, CD34+, Glypican-3 focal+, TTF-1+	CK7+, CK19+, CD34+, Glypican-3+, TTF-1+

HCC, hepatocellular carcinoma; CC, cholangiocarcinoma; APHE, arterial phase hyperenhancement; AFP, alpha-fetoprotein; CA, carbohydrate antigen; CEA, carcinoembryonic antigen; CK, cytokeratin; CD, cluster of differentiation; TTF, thyroid transcription factor.

## Data Availability

Raw data supporting the conclusions of this article will be made available by the authors on request.

## Abbreviations

The following abbreviations are used in this manuscript:

ADC	Apparent diffusion coefficient
AFP	Alpha-fetoprotein
APHE	Arterial phase hyperenhancement
CA 19-9	Carbohydrate antigen 19-9
CEA	Carcinoembryonic antigen
cHCC-CC	Combined hepatocellular–cholangiocarcinoma
CT	Computed tomography
DWI	Diffusion-weighted imaging
ECOG	Eastern Cooperative Oncology Group
HBP	Hepatobiliary phase
iCC	Intrahepatic cholangiocarcinoma
LI-RADS	Liver Imaging Reporting and Data System
MRI	Magnetic resonance imaging
MWA	Microwave ablation
TACE	Transarterial chemoembolization

## References

[1] Duffell E., Pisana A., Stepien M. Liver Cancer Inequalities in Europe and the Role of Viral Hepatitis. https://publications.jrc.ec.europa.eu/repository/handle/JRC138213.

[2] McGlynn K.A., Petrick J.L., Groopman J.D. (2024). Liver Cancer: Progress and Priorities. Cancer Epidemiol. Biomark. Prev. Publ. Am. Assoc. Cancer Res. Cosponsored Am. Soc. Prev. Oncol..

[3] Rumgay H., Arnold M., Ferlay J., Lesi O., Cabasag C.J., Vignat J., Laversanne M., McGlynn K.A., Soerjomataram I. (2022). Global Burden of Primary Liver Cancer in 2020 and Predictions to 2040. J. Hepatol..

[4] Roßner F., Sinn B.V., Horst D. (2023). Pathology of Combined Hepatocellular Carcinoma-Cholangiocarcinoma: An Update. Cancers.

[5] Beaufrère A., Calderaro J., Paradis V. (2021). Combined Hepatocellular-Cholangiocarcinoma: An Update. J. Hepatol..

[6] Terashima T., Harada K., Yamashita T. (2025). Diagnosis, Clinical Characteristics, and Treatment of Combined Hepatocellular-Cholangiocarcinoma. Jpn. J. Clin. Oncol..

[7] Rumgay H., Ferlay J., de Martel C., Georges D., Ibrahim A.S., Zheng R., Wei W., Lemmens V.E.P.P., Soerjomataram I. (2022). Global, Regional and National Burden of Primary Liver Cancer by Subtype. Eur. J. Cancer.

[8] Gera S., Ettel M., Acosta-Gonzalez G., Xu R. (2017). Clinical Features, Histology, and Histogenesis of Combined Hepatocellular-Cholangiocarcinoma. World J. Hepatol..

[9] Eschrich J., Kobus Z., Geisel D., Halskov S., Roßner F., Roderburg C., Mohr R., Tacke F. (2023). The Diagnostic Approach towards Combined Hepatocellular-Cholangiocarcinoma-State of the Art and Future Perspectives. Cancers.

[10] Wang Y., Yang Q., Li S., Luo R., Mao S., Shen J. (2019). Imaging Features of Combined Hepatocellular and Cholangiocarcinoma Compared with Those of Hepatocellular Carcinoma and Intrahepatic Cholangiocellular Carcinoma in a Chinese Population. Clin. Radiol..

[11] Li R., Yang D., Tang C.-L., Cai P., Ma K.-S., Ding S.-Y., Zhang X.-H., Guo D.-Y., Yan X.-C. (2016). Combined Hepatocellular Carcinoma and Cholangiocarcinoma (Biphenotypic) Tumors: Clinical Characteristics, Imaging Features of Contrast-Enhanced Ultrasound and Computed Tomography. BMC Cancer.

[12] Gigante E., Ronot M., Bertin C., Ciolina M., Bouattour M., Dondero F., Cauchy F., Soubrane O., Vilgrain V., Paradis V. (2019). Combining Imaging and Tumour Biopsy Improves the Diagnosis of Combined Hepatocellular-Cholangiocarcinoma. Liver Int. Off. J. Int. Assoc. Study Liver.

[13] Takahashi Y., Dungubat E., Kusano H., Ganbat D., Tomita Y., Odgerel S., Fukusato T. (2021). Application of Immunohistochemistry in the Pathological Diagnosis of Liver Tumors. Int. J. Mol. Sci..

[14] Na H.Y., Kim J.H., Kim H., Cho J.Y., Han H.-S., Jang E.S., Kim J.-W., Jeong S.-H., Heo J., Kim J.-W. (2024). Multiregional Analysis of Combined Hepatocellular-Cholangiocarcinoma Reveals Histologic Diversity and Molecular Clonality. Histopathology.

[15] Jeon Y., Kwon S.M., Rhee H., Yoo J.E., Chung T., Woo H.G., Park Y.N. (2023). Molecular and Radiopathologic Spectrum between HCC and Intrahepatic Cholangiocarcinoma. Hepatology.

[16] Xue R., Chen L., Zhang C., Fujita M., Li R., Yan S.-M., Ong C.K., Liao X., Gao Q., Sasagawa S. (2019). Genomic and Transcriptomic Profiling of Combined Hepatocellular and Intrahepatic Cholangiocarcinoma Reveals Distinct Molecular Subtypes. Cancer Cell.

[17] Gurzu S., Szodorai R., Jung I., Banias L. (2024). Combined Hepatocellular-Cholangiocarcinoma: From Genesis to Molecular Pathways and Therapeutic Strategies. J. Cancer Res. Clin. Oncol..

[18] Yin X., Zhang B.-H., Qiu S.-J., Ren Z.-G., Zhou J., Chen X.-H., Zhou Y., Fan J. (2012). Combined Hepatocellular Carcinoma and Cholangiocarcinoma: Clinical Features, Treatment Modalities, and Prognosis. Ann. Surg. Oncol..

[19] Wakizaka K., Yokoo H., Kamiyama T., Ohira M., Kato K., Fujii Y., Sugiyama K., Okada N., Ohata T., Nagatsu A. (2019). Clinical and Pathological Features of Combined Hepatocellular-Cholangiocarcinoma Compared with Other Liver Cancers. J. Gastroenterol. Hepatol..

[20] Singal A.G., Llovet J.M., Yarchoan M., Mehta N., Heimbach J.K., Dawson L.A., Jou J.H., Kulik L.M., Agopian V.G., Marrero J.A. (2023). AASLD Practice Guidance on Prevention, Diagnosis, and Treatment of Hepatocellular Carcinoma. Hepatol. Baltim. Md.

[21] Choi S.H., Lee S.S., Park S.H., Kim K.M., Yu E., Park Y., Shin Y.M., Lee M.-G. (2019). LI-RADS Classification and Prognosis of Primary Liver Cancers at Gadoxetic Acid-Enhanced MRI. Radiology.

[22] Potretzke T.A., Tan B.R., Doyle M.B., Brunt E.M., Heiken J.P., Fowler K.J. (2016). Imaging Features of Biphenotypic Primary Liver Carcinoma (Hepatocholangiocarcinoma) and the Potential to Mimic Hepatocellular Carcinoma: LI-RADS Analysis of CT and MRI Features in 61 Cases. AJR Am. J. Roentgenol..

[23] Lee S., Kim Y.-Y., Shin J., Son W.J., Roh Y.H., Choi J.-Y., Sirlin C.B., Chernyak V. (2023). Percentages of Hepatocellular Carcinoma in LI-RADS Categories with CT and MRI: A Systematic Review and Meta-Analysis. Radiology.

[24] Nolasco F., Fonseca G.M., de Mello E.S., Kruger J.A.P., Jeismann V.B., Makdissi F.F., Coelho F.F., Alves V.A.F., Herman P. (2025). Prognostic Impact of the Cholangiolar Component in Combined Hepatocellular-Cholangiocarcinoma: Insights from a Western Single-Center Study. J. Surg. Oncol..

[25] Yen C.-C., Yen C.-J., Shan Y.-S., Lin Y.-J., Liu I.-T., Huang H.-Y., Yeh M.M., Chan S.-H., Tsai H.-W. (2021). Comparing the Clinicopathological Characteristics of Combined Hepatocellular-Cholangiocarcinoma with Those of Other Primary Liver Cancers by Use of the Updated World Health Organization Classification. Histopathology.

[26] Chen P.-D., Chen L.-J., Chang Y.-J., Chang Y.-J. (2021). Long-Term Survival of Combined Hepatocellular-Cholangiocarcinoma: A Nationwide Study. Oncologist.

[27] Amory B., Goumard C., Laurent A., Langella S., Cherqui D., Salame E., Barbier L., Soubrane O., Farges O., Hobeika C. (2024). Combined Hepatocellular-Cholangiocarcinoma Compared to Hepatocellular Carcinoma and Intrahepatic Cholangiocarcinoma: Different Survival, Similar Recurrence: Report of a Large Study on Repurposed Databases with Propensity Score Matching. Surgery.

[28] Matsubara K., Kobayashi T., Tadokoro T., Namba Y., Fukuhara S., Oshita K.O., Honmyo N., Kuroda S., Arihiro K., Ohdan H. (2024). The Dominant Component and Clinicopathological Characteristics of Combined Hepatocellular-Cholangiocarcinoma After Radical Resection. Anticancer Res..

[29] Lv T.-R., Hu H.-J., Ma W.-J., Liu F., Jin Y.-W., Li F.-Y. (2024). Meta-Analysis of Prognostic Factors for Overall Survival and Disease-Free Survival among Resected Patients with Combined Hepatocellular Carcinoma and Cholangiocarcinoma. Eur. J. Surg. Oncol. J. Eur. Soc. Surg. Oncol. Br. Assoc. Surg. Oncol..

[30] Liver Imaging Reporting and Data System (LI-RADS) Version 2018: Imaging of Hepatocellular Carcinoma in At-Risk Patients | Radiology. https://pubs.rsna.org/doi/abs/10.1148/radiol.2018181494?journalCode=radiology.

[31] Park J.H., Chung Y.E., Seo N., Choi J.-Y., Park M.-S., Kim M.-J. (2021). Hepatobiliary Phase Signal Intensity: A Potential Method of Diagnosing HCC with Atypical Imaging Features among LR-M Observations. PLoS ONE.

[32] Harper K.C., Ronot M., Wells M.L., Luna A., Ba-Ssalamah A., Wang J., Welle C.L., Silva A.C., Fidler J., Venkatesh S.K. (2025). Hypointense Findings on Hepatobiliary Phase MR Images. Radiogr. Rev. Publ. Radiol. Soc. N. Am. Inc.

[33] Wu H., Liang Y., Wang Z., Tan C., Yang R., Wei X., Jiang X. (2023). Optimizing CT and MRI Criteria for Differentiating Intrahepatic Mass-Forming Cholangiocarcinoma and Hepatocellular Carcinoma. Acta Radiol..

[34] Choi S.H., Lee S.S., Kim S.Y., Park S.H., Park S.H., Kim K.M., Hong S.-M., Yu E., Lee M.-G. (2017). Intrahepatic Cholangiocarcinoma in Patients with Cirrhosis: Differentiation from Hepatocellular Carcinoma by Using Gadoxetic Acid–Enhanced MR Imaging and Dynamic CT. Radiology.

[35] Kim D.H., Choi S.H., Kim D.W., Lee S.S., Lim Y.-S., Kim S.Y., Kim H.J., Kim J.H., Byun J.H. (2021). Combined Hepatocellular-Cholangiocarcinoma: Magnetic Resonance Imaging Features and Prognosis According to Risk Factors for Hepatocellular Carcinoma. J. Magn. Reson. Imaging JMRI.

[36] Jiang H., Song B., Qin Y., Chen J., Xiao D., Ha H.I., Liu X., Oloruntoba-Sanders O., Erkanli A., Muir A.J. (2021). Diagnosis of LI-RADS M Lesions on Gadoxetate-Enhanced MRI: Identifying Cholangiocarcinoma-Containing Tumor with Serum Markers and Imaging Features. Eur. Radiol..

[37] Liu X., Tan S.B.M., Awiwi M.O., Jang H.-J., Chernyak V., Fowler K.J., Shaaban A.M., Sirlin C.B., Furlan A., Marks R.M. (2023). Imaging Findings in Cirrhotic Liver: Pearls and Pitfalls for Diagnosis of Focal Benign and Malignant Lesions. Radiogr. Rev. Publ. Radiol. Soc. N. Am. Inc.

[38] Brown Z.J., Tsilimigras D.I., Ruff S.M., Mohseni A., Kamel I.R., Cloyd J.M., Pawlik T.M. (2023). Management of Hepatocellular Carcinoma: A Review. JAMA Surg..

[39] Benson A.B., D’Angelica M.I., Abbott D.E., Anaya D.A., Anders R., Are C., Bachini M., Borad M., Brown D., Burgoyne A. (2021). Hepatobiliary Cancers, Version 2.2021, NCCN Clinical Practice Guidelines in Oncology. J. Natl. Compr. Cancer Netw. JNCCN.

[40] Na S.K., Choi G.H., Lee H.C., Shin Y.M., An J., Lee D., Shim J.H., Kim K.M., Lim Y.-S., Chung Y.-H. (2018). The Effectiveness of Transarterial Chemoembolization in Recurrent Hepatocellular-Cholangiocarcinoma after Resection. PLoS ONE.

[41] Kim J.H., Yoon H.-K., Ko G.-Y., Gwon D.I., Jang C.S., Song H.-Y., Shin J.H., Sung K.-B. (2010). Nonresectable Combined Hepatocellular Carcinoma and Cholangiocarcinoma: Analysis of the Response and Prognostic Factors after Transcatheter Arterial Chemoembolization. Radiology.

[42] Komuta M. (2021). Histological Heterogeneity of Primary Liver Cancers: Clinical Relevance, Diagnostic Pitfalls and the Pathologist’s Role. Cancers.

[43] Kim T.H., Kim H., Joo I., Lee J.M. (2020). Combined Hepatocellular-Cholangiocarcinoma: Changes in the 2019 World Health Organization Histological Classification System and Potential Impact on Imaging-Based Diagnosis. Korean J. Radiol..

[44] Holzner M.L., Tabrizian P., Parvin-Nejad F.P., Fei K., Gunasekaran G., Rocha C., Facciuto M.E., Florman S., Schwartz M.E. (2020). Resection of Mixed Hepatocellular-Cholangiocarcinoma, Hepatocellular Carcinoma, and Intrahepatic Cholangiocarcinoma. Liver Transplant. Off. Publ. Am. Assoc. Study Liver Dis. Int. Liver Transplant. Soc..

[45] Bahra M., Yahyazadeh A. (2023). Surgical Strategies for Combined Hepatocellular-Cholangiocarcinoma (cHCC-CC). Cancers.

[46] Peng S., Dong S.-C., Bai D.-S., Zhang C., Jin S.-J., Jiang G.-Q. (2023). Radiofrequency Ablation versus Liver Resection and Liver Transplantation for Small Combined Hepatocellular-Cholangiocarcinoma Stratified by Tumor Size. Langenbecks Arch. Surg..

[47] Barrow B., Martin Ii R.C.G. (2023). Microwave Ablation for Hepatic Malignancies: A Systematic Review of the Technology and Differences in Devices. Surg. Endosc..

[48] Auer T.A., Collettini F., Segger L., Pelzer U., Mohr R., Krenzien F., Gebauer B., Geisel D., Hosse C., Schöning W. (2023). Interventional Treatment Strategies in Intrahepatic Cholangiocarcinoma and Perspectives for Combined Hepatocellular-Cholangiocarcinoma. Cancers.

[49] Renzulli M., Ramai D., Singh J., Sinha S., Brandi N., Ierardi A.M., Albertini E., Sacco R., Facciorusso A., Golfieri R. (2021). Locoregional Treatments in Cholangiocarcinoma and Combined Hepatocellular Cholangiocarcinoma. Cancers.

[50] Trikalinos N.A., Zhou A., Doyle M.B.M., Fowler K.J., Morton A., Vachharajani N., Amin M., Keller J.W., Chapman W.C., Brunt E.M. (2018). Systemic Therapy for Combined Hepatocellular-Cholangiocarcinoma: A Single-Institution Experience. J. Natl. Compr. Cancer Netw. JNCCN.

[51] Su G.L., Altayar O., O’Shea R., Shah R., Estfan B., Wenzell C., Sultan S., Falck-Ytter Y. (2022). AGA Clinical Practice Guideline on Systemic Therapy for Hepatocellular Carcinoma. Gastroenterology.

[52] Kim E.J., Yoo C., Kang H.J., Kim K.-P., Ryu M.-H., Park S.R., Lee D., Choi J., Shim J.H., Kim K.M. (2021). Clinical Outcomes of Systemic Therapy in Patients with Unresectable or Metastatic Combined Hepatocellular-Cholangiocarcinoma. Liver Int. Off. J. Int. Assoc. Study Liver.

[53] Azizi A.A., Hadjinicolaou A.V., Goncalves C., Duckworth A., Basu B. (2020). Update on the Genetics of and Systemic Therapy Options for Combined Hepatocellular Cholangiocarcinoma. Front. Oncol..

[54] Nguyen C.T., Caruso S., Maille P., Beaufrère A., Augustin J., Favre L., Pujals A., Boulagnon-Rombi C., Rhaiem R., Amaddeo G. (2022). Immune Profiling of Combined Hepatocellular- Cholangiocarcinoma Reveals Distinct Subtypes and Activation of Gene Signatures Predictive of Response to Immunotherapy. Clin. Cancer Res. Off. J. Am. Assoc. Cancer Res..

[55] Faivre S., Rimassa L., Finn R.S. (2020). Molecular Therapies for HCC: Looking Outside the Box. J. Hepatol..

